# User Experience of a Large-Scale Smartphone-Based Observational Study in Multiple Sclerosis: Global, Open-Access, Digital-Only Study

**DOI:** 10.2196/57033

**Published:** 2024-09-11

**Authors:** Adriano Galati, Lito Kriara, Michael Lindemann, Rea Lehner, JB Jones

**Affiliations:** 1 F Hoffmann-La Roche Ltd Basel Switzerland; 2 Sutter Health Center for Health Systems Research Walnut Creek, CA United States

**Keywords:** smartphone, multiple sclerosis, user experience, retention, digital health, digital health technology, mobile phone

## Abstract

**Background:**

The Floodlight Open app is a digital health technology tool (DHTT) that comprises remote, smartphone sensor–based tests (*daily activities*) for assessing symptoms of multiple sclerosis (MS). User acquisition, engagement, and retention remain a barrier to successfully deploying such tools.

**Objective:**

This study aims to quantitatively and qualitatively investigate key user experience (UX) factors associated with the Floodlight Open app.

**Methods:**

Floodlight Open is a global, open-access, digital-only study designed to understand the drivers and barriers in deploying a DHTT in a naturalistic setting without supervision and onboarding by a clinician. Daily activities included tests assessing cognition (Information Processing Speed and Information Processing Speed Digit–Digit), hand-motor function (Pinching Test and Draw a Shape Test), and postural stability and gait (Static Balance Test, U-Turn Test, and Two-Minute Walk Test [2MWT]). All daily activities except the 2MWT were taken in a fixed sequence. Qualitative UX was studied through semistructured interviews in a substudy of US participants with MS. The quantitative UX analysis investigated the impact of new UX design features on user engagement and retention in US participants for 3 separate test series: all daily activities included in the fixed sequence (DA), all daily activities included in the fixed sequence except the Static Balance Test and U-Turn Test (DA_x_), and the 2MWT.

**Results:**

The qualitative UX substudy (N=22) revealed the need for 2 new UX design features: a more seamless user journey during the activation process that eliminates the requirement of switching back and forth between the app and the email that the participants received upon registration, and configurable reminders and push notifications to help plan and remind the participants to complete their daily activities. Both UX design features were assessed in the quantitative UX analysis. Introducing the more seamless user journey (original user journey: n=608; more seamless user journey: n=481) improved the conversion rate of participants who enrolled in the study and proceeded to successfully activate the app from 53.9% (328/608) to 74.6% (359/481). Introducing reminders and push notifications (with reminders and notifications: n=350; without reminders and notifications: n=172) improved continuous usage time (proportion of participants with ≥3 consecutive days of usage: DA and DA_x_: ~30% vs ~12%; 2MWT: ~30% vs ~20%); test completion rates (maximum number of test series completed: DA: 279 vs 64; DA_x_: 283 vs 126; 2MWT: 302 vs 76); and user retention rates (at day 30: DA: 53/172, 30.8% vs 34/350, 9.7%; DA_x_: 53/172, 30.8% vs 60/350, 17.1%; 2MWT: 39/172, 22.6% vs 22/350, 6.2%). Inactivity times remained comparable.

**Conclusions:**

The remote assessment of MS with DHTTs is a relatively nascent but growing field of research. The continued assessment and improvement of UX design features can play a crucial role in the successful long-term adoption of new DHTTs.

## Introduction

### Background

Traditionally, multiple sclerosis (MS) has been categorized as having a relapsing-remitting or progressive course. However, recent work shed light on an underlying insidious progression, or a progression that is independent of relapses, even in patients previously thought to have a relapsing-remitting disease [1]. Therefore, minimizing or even eliminating progression is one of the goals of MS disease management. To achieve this goal, sensitive measures of MS-related functional ability are needed that can be frequently administered with minimal burden to the patient [2]. Here, digital health technology tools (DHTTs) such as smartphone sensor–based tests offer a new, promising strategy [3-5]. By taking advantage of the large variety of embedded sensors, smartphones enable the remote assessment of several functional domains affected by MS without supervision by a clinician [3]. However, user retention remains a barrier to the successful deployment of such DHTTs [6].

Assessments of user experience (UX) should, therefore, be considered when designing DHTTs [7]. They have attracted increasing attention, as they provide important insights to enhance usability, engagement, perception, and satisfaction [8-11]. With different UX designs available, it is important to keep in mind that their effectiveness depends on both the intended users and the environment in which they are being used. Hence, a comprehensive assessment of UX should include the study of both qualitative and quantitative UX in different environments for an extended period. The primary focus of qualitative UX assessments is to characterize the individual user’s experiences with the DHHT and to elucidate fine-grained aspects of why and how users engage with it [12-14]. Quantitative UX assessments, by comparison, provide strong indicators of the duration and frequency of user engagement and retention and are typically conducted in larger cohorts.

### Aims

**Figure 1:**
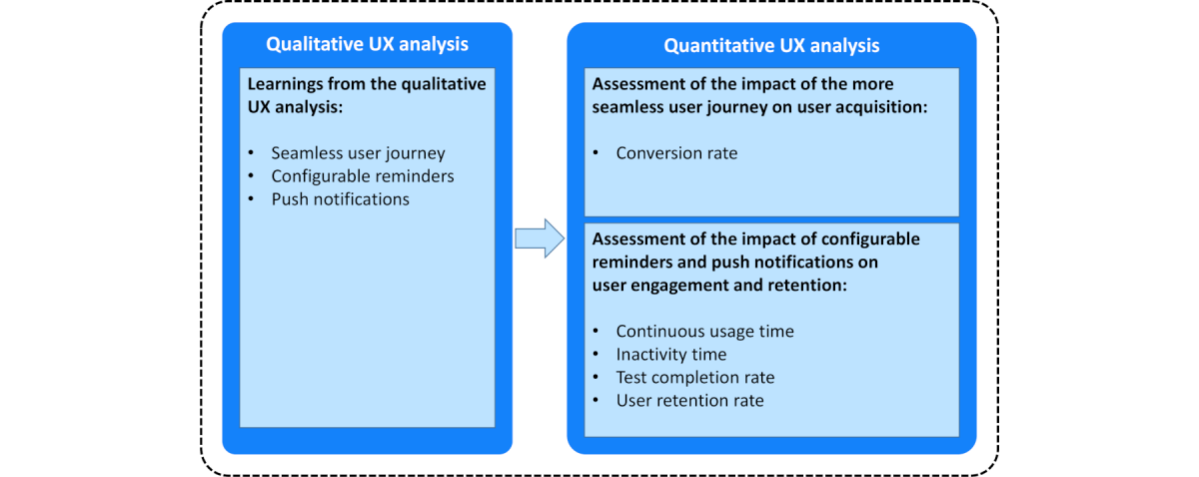
Learnings from the quantitative user experience (UX) analysis led to the implementation of new UX design features, such as a more seamless user journey from registration through the activation of the Floodlight Open app, configurable reminders, and push notifications. The impact of these new UX design features was assessed in the quantitative UX analysis.

Here, we present a qualitative and quantitative UX analysis of the Floodlight Open app (Figure 1). The app comprises patient-reported outcomes and smartphone sensor–based tests that assess mood, cognition, hand-motor function, postural stability and gait, and mobility levels [15]. It was deployed in Floodlight Open, a global, open-access, digital-only study that was designed to understand the drivers and barriers in the deployment of the app in a naturalistic setting without supervision and onboarding by a clinician, in a broad, multinational study cohort [15]. The UX analyses presented here allow us to gain a better understanding of the participants’ behavior with the Floodlight Open app. This will help make informed UX design decisions to improve engagement with the Floodlight technology. Qualitative UX is assessed through semistructured interviews conducted with a subset of US participants with MS [16] to identify important elements that could improve UX and user engagement. In addition, quantitative UX is assessed by evaluating the impact of UX design changes, which were motivated by the learnings from the qualitative UX analysis, on user engagement and retention using data collected from Floodlight Open.

## Methods

### Participants and Study Design

Floodlight Open’s full study design and inclusion and exclusion criteria have been previously reported [15]. Adults with or without MS who owned a suitable iOS (Apple Inc) or Android (Google LLC) smartphone and resided in 1 of the 17 participating countries were permitted to take part. The MS status was self-declared by the participants. To join Floodlight Open, participants first registered on the Floodlight Open web portal and completed the informed e-consent process. After successfully completing these 2 steps, each participant received their personal Floodlight Open unique identifier (FLO ID) and an activation code (ie, an activation token [15]) via email. Both were required to activate the Floodlight Open app on the participant’s personal smartphone device. The email also provided a link to the Floodlight Open web portal, where participants could request a new activation code (the activation code was valid for 48 hours, after which it expired), access their account to update their profile, view their results, or withdraw from the study.

**Figure 2:**
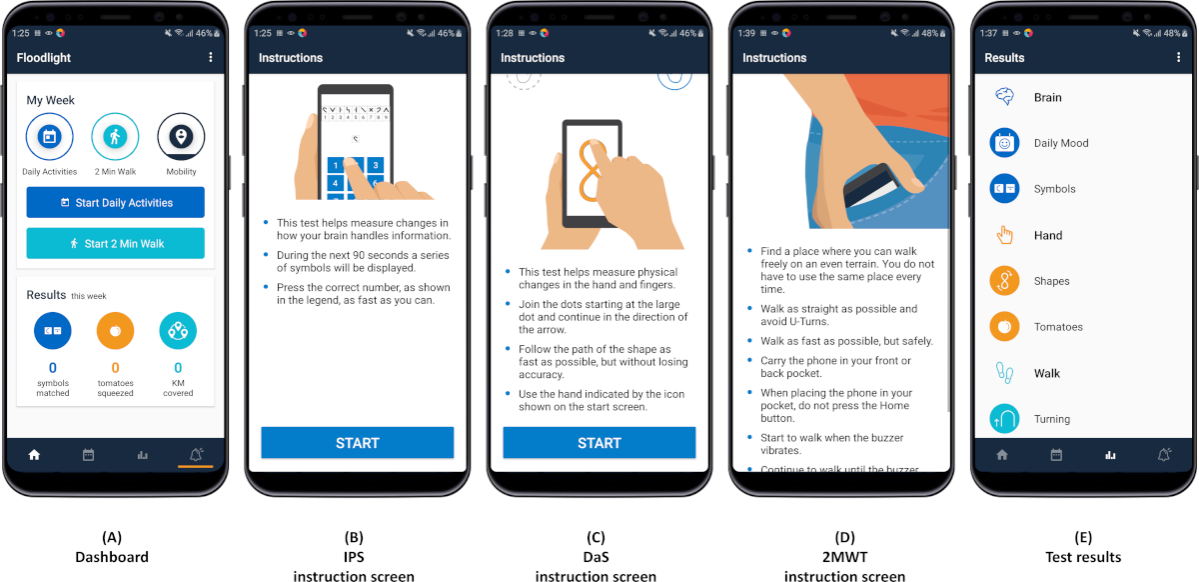
Screenshots from the Floodlight Open app. The dashboard (A) enables participants to set reminders by tapping the bell-shaped reminder icon in the bottom right corner. Participants can self-administer smartphone–based tests to assess cognition (Information Processing Speed [IPS; B] and IPS Digit–Digit), hand-motor function (Pinching Test and Draw a Shape Test [DaS; C]), and gait and postural stability (Static Balance Test, U-Turn Test, and 2-Minute Walk Test [2MWT; D]). The results of these tests are provided on the results screen (E).

After the activation of the Floodlight Open app, several tests, or assessments, were made available to the participants. These included a patient-reported outcome assessing the participants’ mood (Daily Mood Questionnaire [DMQ] [15]) and a series of smartphone sensor–based active tests (ie, tests that require active input from the user) measuring cognition (Information Processing Speed [IPS] [15] and IPS Digit–Digit [15]); hand-motor function (Pinching Test [PT] [15,17] and Draw a Shape Test [DaS] [15]); and postural stability and gait (Static Balance Test [SBT] [15], U-Turn Test [UTT] [15,18], and Two-Minute Walk Test [2MWT] [15]; Figure 2).

In addition, data on mobility level were passively collected without requiring input from the user (*passive monitoring*) through life-space measurement [15], which measured the distance between the 2 farthest GPS coordinates detected during the day that were at least 500 m apart, resulting in 1 measurement per day. Since this passive monitoring does not require any active input from the user, it is not included in our UX analyses.

The DMQ and all active tests except the 2MWT were administered in a predefined, fixed sequence comprising the daily activities. The DMQ was administered first, followed by the IPS, IPS Digit–Digit, PT, DaS, SBT, and UTT. The 2MWT was administered separately from these daily activities, as this test required the participant to walk in a straight line for 2 minutes, which may not always be possible at the time of taking the other active tests. The tests could be taken up to once a week (IPS) or once a day (all other tests), but the actual test frequency was not systematically enforced. For example, the UTT, SBT, and 2MWT could be skipped if participants determined that the conditions for safe execution, including environmental and physical factors, could not be met that day.

### Ethical Considerations

Data collected with the Floodlight Open app were encrypted and electronically stored on 2 specific, secure cloud databases, which were made publicly available for the duration of the study and maintained by the study initiator. To ensure confidentiality, all participant information was pseudonymized through association with the personal FLO ID. Furthermore, no personal identifiable information was collected while the participants executed the Floodlight Open tests. This meant that GPS coordinates were obfuscated and excluded from the public data set to protect participants’ privacy.

The protocol, informed e-consent forms, and relevant supporting information were reviewed and approved by the appointed central institutional review boards or ethics committees before the study was initiated in each participating country, as applicable, in accordance with each country’s regulatory requirements [15]. The institutional review board for the United States was the Western Institutional Review Board in Puyallup, Washington (approval: 20180617).

### Qualitative UX Analysis

The qualitative UX substudy was conducted by a health care delivery network (Sutter Health) in a subset of US participants with MS who took part in Floodlight Open and agreed to download the app and use it daily for 30 days [16]. This substudy aimed at elucidating finer-grained aspects of why and how users engage with the Floodlight Open app in a particular manner and at providing information on UX design features that could improve UX and user engagement. Individual, semistructured interviews were conducted to gain insights into the participants’ experiences with the Floodlight Open app and the perceived benefits thereof (Table 1). The use of individual interviews offered privacy and enabled the exploration of each participant’s interaction with the app. A multifaceted recruitment strategy was applied to involve participants of a broad age range to combine the experiences of the tech-savvy younger generation with that of the older generation. Most participants were using their smartphone and their computer daily. No preferential sampling of participants with either negative or positive experiences was applied. Any duration of use of the Floodlight Open app was of value because discontinued use and negative experiences can provide valuable insights and complement data from persistent participants.

**Table 1:** Interview guide for the qualitative UX^a^ analysis.

Question			Elaboration questions
**Technology acceptance**			
	Were you able to download the app to your phone and get it working?	How was that process? Was there anything you found particularly easy, or hard or confusing about the process?	
	We asked people taking part in the study to use the app for 30 days. For about how many days did you use the app?	0, 1-7, 8-14, 14-21, 22-29, 30+, or don’t remember If answer is “0” or “30+,” skip the next question.	
	What made you decide to either not use it or stop using it after less than 30 days?	Probe to identify root cause and any efforts made to overcome: App related (technical problems, overall demand in terms of time or frequency, or understandability) Phone related (data, service, or access) User related (general willingness, utility, or physical or emotional ability)	
	Before you started using the app, did you feel like you had a good idea about what it was designed to do?	Did you have any questions or concerns? What were they?	
**Experience of use**			
	How well could you see the information on the screen?	Was there anything that was hard for you to see?	
	How easy was it to understand the information and prompts?	Was there anything that was hard to understand?	
	How well did using the app fit into your life, such as the timing and frequency of prompts, and time it took to respond?	Not applicable	
**Perception**			
	Did tracking information about MS^b^ on a regular basis using this app teach you anything new about your health or give you a better sense of how you’re doing?	Why or why not? Did you do/think/feel anything different based on what you learned?	
	Do you think this is an application you can use on daily basis?	Why or why not? If not daily, what do you think is the optimal frequency of use?	
	How would you feel about being prompted by the app on a daily basis to do tasks or answer questions related to MS?	If not daily, what do you think is the optimal frequency of reminders and notification?	
	Did you share the information from the app (either directly or what you learned) with anyone else?	Do you mind telling me what you shared?	
	Of the information collected by the app, what would you want to share with your doctor?	What is valuable about this information? How would sharing it impact your appointment in terms of your discussions, care or treatment?	
	Is there anything else you would like to add about your experience tracking MS on a daily basis using your phone?	Not applicable	
**General health questions: disease severity and duration**			
	Before we end our interview I have a few general questions about your health and computer use. What year were you diagnosed with MS?	Not applicable	
	What type of MS do you have at this time?	Relapsing-Remitting MS (RRMS) Secondary-Progressive MS (SPMS) Primary-Progressive MS (PPMS) Progressive-Relapsing MS (PRMS)	
**Technology literacy**			
	Last question. I’ll give you the question and then I’ll read a list of options for your response. How often do you use a computer?	Everyday At least once a week but not everyday Less than once a week but more than once a month Less than once a month Never	

^a^UX: user experience.

^b^MS: multiple sclerosis.

Semistructured interviews were conducted via phone by a trained interviewer, with 2 attempts made to reach each participant. The interviews lasted between 15 and 20 minutes and were audiotaped and fully transcribed for further analysis. A reductionist approach was applied to deconstruct implicit and explicit responses into manageable variables. The qualitative paradigm was crucial to appreciating, observing, and deducing participants’ experiences [19]. Through an inductive analysis of the data collected, the common needs of the participants were identified, which should be considered to improve the UX and to develop preliminary conclusions on the need for specific UX features.

### Quantitative UX Analysis

#### Participants

The quantitative UX analysis was conducted in all US participants who enrolled in Floodlight Open [15]. The analysis was restricted to US participants for several reasons. First, information on user acquisition, including the date and time of activation of the FLO ID, was collected only in the United States. Second, the release dates of the iOS and Android versions of the Floodlight Open app differed for each country [15]. Due to these different release dates, only the United States offered a sufficiently long period during which the app’s use without the new UX design features could be studied. Finally, limiting the analysis to US participants allowed us to keep the quantitative UX analysis as consistent as possible with the qualitative UX analysis, which was conducted in a subset of the US participants, thus reducing the impact of differences in demographics and user behavior between the 2 analyses.

#### Implementation of New UX Design Features

**Figure 3:**
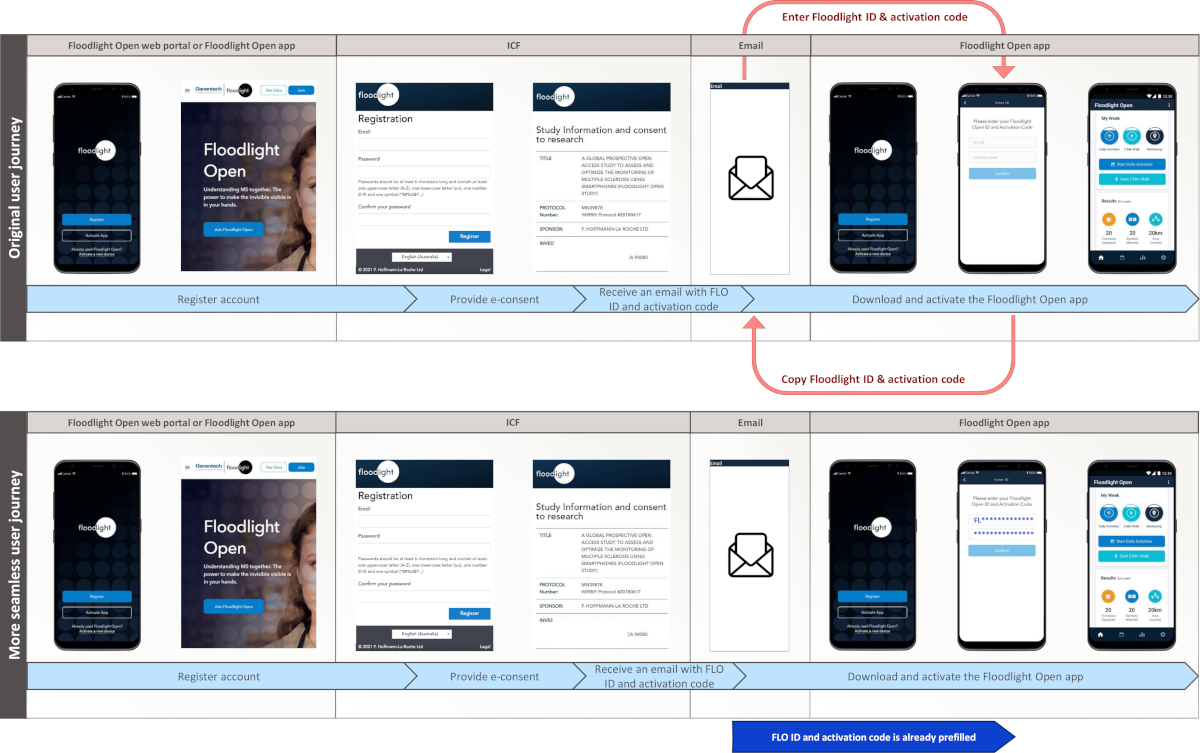
User journey from creating an account to activating the Floodlight Open app. This user journey consists of 4 steps: creating an account, providing e-consent, receiving a personalized Floodlight Open unique identifier (FLO ID) and activation code via email, and downloading and activating the Floodlight Open app (light blue arrows). With the original user journey, participants will have to switch between the app and the email at least twice to copy and enter their FLO ID and activation code to activate all functionalities of the app (red arrows). With the more seamless user journey, the deep-linking feature is integrated in the link provided in the email to download the app from the local app store. With this feature, the personalized FLO ID and activation code are prefilled when the participants launch the Floodlight Open app for the first time (dark blue arrow). Consequently, participants do not have to switch back and forth between interfaces (ie, their email and the Floodlight Open app) to activate all functionalities. ICF: informed e-consent form.

The quantitative UX analysis was conducted to investigate the impact of the new UX design features identified in the qualitative UX analysis on user engagement and retention. One of the new UX design features identified in the qualitative UX analysis was a more seamless user journey, which guides users from registration to the activation of the Floodlight Open app (Figure 3). The original user journey involved registering on the Floodlight Open web portal; downloading the Floodlight Open app from the local app store; and activating the app by entering the FLO ID and activation code, which the participants received in a notification email. This last step required participants to switch multiple times between the notification email and the Floodlight Open app to manually enter their FLO ID and activation code (original user journey in Figure 3). These complex interactions with multiple user interfaces may frustrate participants, who may eventually abandon the activation process. Therefore, to minimize nuisance factors and reduce user loss, a more seamless user registration and app activation journey was implemented by deep linking the notification email with the Floodlight Open app. This allowed participants to launch the Floodlight Open app directly from the notification email, if already installed on their phone, or to download it from their local app store, with the fields for the FLO ID and activation code already filled out (ie, deep linked). Thus, this more seamless user journey eliminated the requirement of searching for the FLO ID and activation code in the notification email and manually inserting them in the right fields in the Floodlight Open app to activate it (more seamless user journey in Figure 3).

In addition, configurable reminders and push notifications were introduced as the second new UX design feature (Table 2). Configurable reminders provided participants the option to set up reminders to perform the tests. Participants could set up daily or weekly reminders by tapping on the reminder icon, which was visible on the app’s dashboard (Figure 2). Push notifications, by comparison, were designed to serve two purposes: (1) to encourage inactive participants to reengage with the Floodlight Open app, for example, when they closed the app and did not complete the tests or have been inactive for some time, and (2) to motivate committed users to keep performing the tests. Tapping on the notification took the participants directly to the right entry point of the Floodlight Open app to perform the uncompleted or next test. To enable this new feature, participants had to grant permission to receive push notifications.

**Table 2:** List of push notifications and configurable reminders implemented in the Floodlight Open app^a^.

Feature and type		Notification	Description
**Push notifications**			
	Authorization request	Floodlight would like to send you notifications. We’ll send you reminders and alerts to keep you on top of your goals and up-to-date with the impact you are having. [Don’t Allow | Ok]	Sent the first time users open the app Permission request to allow push notifications: the Floodlight Open app can send notifications to users at different touch points within the users’ journey in the app User declines permission: the Floodlight Open app cannot send notifications to users. This affects the user experience
	Authorization reminder	Need help keeping up with your Daily Activities? You can allow notifications in your Settings. [Go to Settings | Not now]	Sent if permission is declined and when the app is open for the first time after 7 days, but never during an active test The Go to Settings button opens the Floodlight screen in the phone settings, where users can re-enable notifications
	Authorization accepted	Thanks for downloading Floodlight Open. Let’s get you signed up and started!	If accepted, it opens immediately on the sign-up screen
	Incomplete daily activities	You’re doing great! Do you have time to [log your mood | start on symbols | squash some tomatoes | see the shapes | handle balance | get moving]?	When users do not complete daily activities in one go, a notification is sent in 4 hours or at 7 PM, at the latest. No notifications are sent after 7 PM, and notifications are sent no more than once per day Tapping on the notification takes users to the next test in the daily activities to continue with the remaining tests. The message is dependent on the last test completed Multiple notifications are never sent
	Completed only daily activities	You have finished your Daily Activities – ready to finish up with a 2MWT^b^?	When users complete only daily activities but not the 2-minute walk, a notification is sent in 4 hours or at 7 PM, at the latest. No notifications are sent after 7 PM, and notifications are sent no more than once per day Clicking on the notification takes users to the 2MWTb
	Incomplete 2MWT^b^	Don’t forget your 2MWT^b^ today. If you can, get started today!	When users access and abandon the 2MWTb, a notification is sent after 4 hours or at 7 PM, at the latest. No notifications are sent after 7 PM, and notifications are sent no more than once per day Tapping on the notification takes users to the 2-minute walk Users receive this reminder every time the 2MWTb is abandoned
	Completed only the 2MWT^b^	You’ve checked off your 2MWT^b^ – now let’s finish up with your Daily Activities.	When users complete only the 2MWTb but not daily activities, a notification is sent in 4 hours or at 7 PM, at the latest. No notifications are sent after 7 PM, and notifications are sent no more than once per day Tapping on the notification takes users to the daily activities Users receive this reminder only once a day
	Encouragement	You’ve done an incredible job helping us build research so far – ready to check another day off your list?	Sent at 10 AM the day after 3 consecutive test runs are completed Tapping on the notification takes users to the daily activities Users receive this notification no more than twice per week
	Encouragement	It’s that time again – ready to check another day off your list?	Sent at 10 AM the day after 3 consecutive days of no test runs Tapping on the notification takes users to the dashboard
	Acknowledgment	Just a quick thank you. Your support is helping us learn more about MS. We hope you find it useful too!	Sent every 2 weeks at 6 PM Tapping on the notification takes users to the dashboard
**Configurable reminders**			
	General reminder	It’s time to complete my Daily Activities and 2MWT^b^. Let’s do it!	Only 1 reminder can be set up, which can be triggered daily, weekly, or on certain days of the week and at any time

^a^Push notifications and configurable reminders were made available on October 21, 2019, for the iOS version and on February 19, 2020, for the Android version of the Floodlight Open app.

^b^2MWT: Two-Minute Walk Test.

The push notifications were designed as a friendly communication with a strong emphasis on the emotional context (Table 2), as the emotional interpretation of a message can impact the user’s response [20,21]. The emotional interpretation begins with the perception of a friendly communication and leads to the planning and execution of a responsive action. Notifications designed in this way are less likely to be ignored and can help create the perception of a human touch within the Floodlight Open app. Hence, they are quite effective for eliciting short-term actions [22] and are an important variable in improving adherence [23].

The frequency and logic with which push notifications were triggered were carefully considered to limit disturbance. Receiving many notifications in a short interval or during inopportune times can overwhelm and irritate users [24-26], which may cause them to turn off notifications or even uninstall the app. Therefore, the notifications were designed such that they are never sent more than twice per day, never sent after 7 PM, and sent only when the user seems to have abandoned the Floodlight Open tests.

#### Statistical Analysis

The quantitative UX analysis was conducted on data available in the publicly available data set [15]. As information on self-declared MS status was collected only after the Floodlight Open app was activated, both participants with MS and participants without MS were included in the analysis on the effectiveness of the more seamless user journey. In contrast, the analysis on configurable reminders and push notifications was limited to participants with MS to keep the analysis as consistent as possible with the quantitative UX analysis.

To study the effectiveness of the more seamless user journey, the conversion rate, which is the proportion of participants who successfully completed the registration and activation process, was compared across 2 cohorts. The first cohort followed the original user journey without the deep-linking feature, whereas the second cohort followed the more seamless user journey that took advantage of the deep-linking feature. Because this UX design feature was released on a separate schedule for the iOS and Android versions of the Floodlight Open app, the first cohort included data collected from November 12, 2018, when the activation dates of the FLO IDs were first logged, through July 17, 2019, when the more seamless user journey along with the deep-linking feature was first implemented. The second cohort included data from all participants who registered from October 21, 2019, when the more seamless user journey was fully implemented in the United States, through November 2, 2021, when the study closed in the United States. The gap between July 17, 2019, and October 21, 2019, was required, as the more seamless user journey feature was released on different schedules for iOS and Android platforms.

The impact of introducing push notifications and configurable reminders on UX (ie, user engagement and retention) was assessed through continuous app usage time, inactivity time, test completion rate, and user retention rate. The continuous usage time is the number of consecutive days on which a participant used the Floodlight Open app, namely, on how many days in a row they performed the Floodlight Open tests.

The inactivity time, by comparison, is the time interval between 2 continuous use times during which a participant did not perform any test, that is, how many days in a row a participant waited before returning to perform a test. This metric is strongly affected by the frequency with which participants performed the tests and the likelihood of participants returning to the tests. The nature of the distribution can guide, for example, the choice of successful engagement methods that can further increase user engagement and retention. For both continuous usage time and inactivity time, the probability of *n* days of continuous usage and inactivity, respectively, was computed.

The test completion rate is defined as the fraction of participants performing at least *n* test series (DA, all daily activities included in the fixed sequence except the SBT and UTT [DA_x_], or 2MWT) since the activation of the Floodlight Open app. The completion rate is a common measure for assessing the effectiveness of UX features, that is, for assessing whether the tasks performed by users achieve specified goals in terms of accuracy and completeness in a specified context of use [27]. It does not consider how the goals were achieved but only the extent to which they were achieved. The higher the test completion rate is, the more engaged the users are and the more likely they are to come back and, in this case, perform the assessments. The test completion rate is calculated over the entire duration of the assessed period and does not distinguish between intermittent and continuous usage.

The user retention rate, by comparison, is the fraction of participants who returned to perform the Floodlight Open tests *n* days after the activation of the Floodlight Open app. The retention rate is extensively used to measure the success of smartphone apps, with higher retention corresponding to higher adoption and level of engagement [28,29].

These UX metrics (continuous app usage time, inactivity time, test completion rate, and user retention rate) characterize the UX with the Floodlight Open app and provide insights into how committed the participants are. Because taking the gait and postural stability tests require both time and space (see the *Qualitative UX Analysis* section), 3 separate test series were considered for each of these metrics: DA (ie, all active tests administered in the fixed sequence: DMQ, IPS, IPS Digit–Digit, PT, DaS, SBT, and UTT), all DA except the gait and postural stability tests (DA_x_; ie, DMQ, IPS, IPS Digit–Digit, PT, and DaS), and 2MWT.

For each test series, the complementary cumulative distributions of the 4 UX metrics were compared in MS participants using data collected before (November 12, 2018, through October 21, 2019) versus after (February 19, 2020, through November 2, 2021) the configurable reminders and push notifications were introduced. The gap between November 12, 2018, and October 21, 2019, was necessary due to the different release schedules of reminders and notifications for the iOS and Android versions. Hence, during this period, reminders and notifications were available to some, but not all, MS participants. It is possible that individual participants were included in both observation periods if they took the Floodlight Open tests during both periods. Because the different durations of these 2 periods might impact the continuous usage times and the inactivity times, both UX metrics were assessed over the first 343 days of data collected from each participant.

## Results

### Participants

Until November 2, 2021, when the study closed in the United States, the Floodlight Open app was downloaded 5225 times, including 4240 (81.15%) times on iOS devices and 985 (18.85%) times on Android devices, across the 17 participating countries. The baseline demographics of the US participants with MS included in the qualitative and quantitative UX analyses on reminders and notifications are presented in Table 3.

**Table 3:** Baseline demographics of US participants with MS^a^ included in the qualitative UX^b^ analysis and the quantitative UX analysis.

Variable			Qualitative UX analysis (N=22^c^)		Quantitative UX analysis^d^, n (%)												
					FLO^e^ app without configurable reminders and push notifications^f^ (n=350)					FLO app with configurable reminders and push notifications^g^ (n=172)					All^h^ (N=498)		
					Female (n=264)		Male (n=86)		Female (n=123)			Male (n=49)		Female (n=368)			Male (n=130)
Age (y), mean (SD; range)			50 (9.9; 18-74)		47.53 (11.4; 19-71)		49.63 (13.2; 19-84)		48.69 (12.29; 22-73)			50.73 (10.57; 30-79)		47.6 (11.6; 19-73)			49.7 (12.7;19-84)
**Age distribution (y), n (%)**																	
	18-24	0 (0)		4 (1.5)		1 (1.2)		4 (3.3)			0 (0)		6 (1.6)			1 (0.8)	
	25-34	0 (0)		32 (12.1)		12 (13.9)		10 (8.1)			3 (6.1)		42 (11.4)			16 (12.3)	
	35-44	3 (13.6)		64 (24.2)		19 (22.1)		32 (26)			11 (22.5)		99 (26.9)			29 (22.3)	
	45-54	7 (31.8)		90 (34.1)		22 (25.6)		31 (25.2)			19 (38.8)		111 (30.2)			38 (29.2)	
	55-64	7 (31.8)		53 (20.1)		24 (27.9)		31 (25.2)			12 (24.5)		78 (21.2)			34 (26.2)	
	65-74	5 (22.7)		21 (8)		7 (8.1)		15 (12.2)			3 (6.1)		32 (8.7)			10 (7.7)	
	≥75	0 (0)		0 (0)		1 (1.2)		0 (0)			1 (2)		0 (0)			2 (1.5)	

^a^MS: multiple sclerosis.

^b^UX: user experience.

^c^Of the 22 participants included in the qualitative user experience (UX) analysis, 19 (86.4%) were female.

^d^Some participants may be included both in the cohort with configurable reminders and notifications and in the cohort without configurable reminders and notifications if they provided data during both periods. Demographic information was collected only after the successful activation of the Floodlight Open app and is, therefore, not available for the participants included in the assessment of the more seamless user journey.

^e^FLO: Floodlight Open.

^f^Includes all US participants with MS who participated in the study from November 12, 2018, through October 20, 2019.

^g^Includes all US participants with MS who participated in the study from February 19, 2020, through November 2, 2021.

^h^Includes all US participants with MS who participated in the study from November 12, 2018, through November 2, 2021, and were included in the analysis of the impact of introducing configurable reminders and push notifications.

### Qualitative UX Analysis

A total of 22 US participants were enrolled in the qualitative substudy, of which 15 (68%) were interviewed. Between 7 and 19 participants are considered adequate for qualitative research [30].

The qualitative UX analysis reveals key points for improving the UX with the Floodlight Open app (Table 4). While many participants did not face any difficulties downloading the Floodlight Open app and were willing to perform the tests daily, some were unaware or confused about the activation process despite instructions being provided on the study’s web portal (see *Need for a More Seamless User Journey* section in Table 4). Other participants reported that they did not complete the activation process due to distractions or the lack of reminders to complete this step. Here, a more seamless journey from registering on the web portal to activating the Floodlight Open app could improve the UX. In addition, participants who successfully activated the Floodlight Open app self-reported that they used it for 2 to 30 days and agreed that screen contents, prompts, and information were easy to see and understand. However, they also reported that they often forgot to perform the tests or felt it was not always feasible to perform all the tests in one setting. Therefore, configurable reminders and push notifications would have been beneficial (see bottom section in Table 4). Such UX design features could be used to set up reminders to perform the tests or to stop the current test and plan to complete the tests later. Furthermore, participants were not always available to perform tests that required them to stand or walk:

I can only proceed so far because I’m in the car waiting for my kid; I can’t stand and balance...or do the walking back and forth and the balancing stuff.

The part that is difficult to get done is the 2-minute walk. To find the time...is sometimes hard for someone like me who is more active.

Certain tasks...were a little too long. How many more lines do I need to draw? How many more tomatoes can I pinch?

**Table 4:** Learnings from the qualitative UX^a^ analysis.

Learnings from the qualitative UX analysis	Comments made by participants that support the learnings
Need for a more seamless user journey	“I see a register button and an activate button, and I don’t know if I am registered, or what I need to do to activate it. I remember getting to this page and thinking there’s something I have to do, some code or something to be able to activate it.” “I let it go too long before I tried to use it, and so the registration/activation process was not clear to me at that point and I never followed up on it.”
Need for configurable reminders and push notifications	“I just don’t know. I think maybe it was something that was on my phone, and I was like, ‘What is this? You know?’” “I would say life got in the way.” “I just forgot. I got distracted. It just went out of my brain and never came back. What I should have done in retrospect is put a reminder popup in my phone, like to pop up every day. I would much rather the app remind me. The less I have to touch my phone, the better my life is.” “I have been terrible at using the app. I’ve used it twice. I think one of the problems I had with the app is it doesn’t remind me to do it. If my phone doesn’t tell me to do something, it probably doesn’t happen. I would have been much more inclined to do it if it had reminded me to do it.” “Things were crazy with work. I’ve had other things on my mind. I didn’t see anything pop up reminding me to use it.” “I didn’t read the instructions. I had to guess what to do with the matching shapes. At first I thought I was being timed so I was rushing.”

^a^UX: user experience.

Consequently, in the subsequent section on quantitative UX analysis, UX with the Floodlight Open app was investigated with respect to DA, DA_x_, and 2MWT.

### Quantitative UX Analysis

#### User Journey

**Figure 4:**
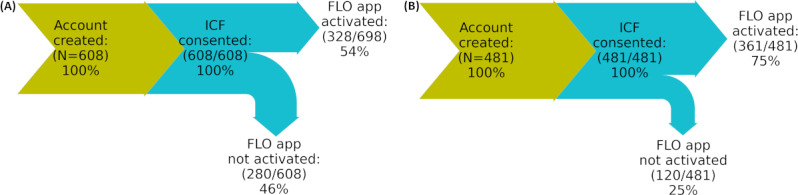
Conversion rate diagrams. These diagrams depict the user journey from registration on the Floodlight Open web portal (creating a Floodlight Open account) to the activation of the Floodlight Open app (A) before and (B) after implementing the more seamless user journey. FLO: Floodlight Open; ICF: informed e-consent form.

Of the 1089 US participants, 608 (55.8%) with or without MS (self-declared MS disease status was not available for this analysis, as this information was collected only after the successful activation of the app) followed the original user journey without the deep-linking feature, and 481 (44.2%) participants with or without MS followed the newer, more seamless user journey with the deep-linking feature. The flow diagrams in Figure 4 show the conversion rates from creating an account to activating the app for both cohorts. In both cohorts, all participants who created an account completed the registration process, provided informed consent, and enrolled in the study. However, Figure 4 shows that introducing the new user journey improved the conversion rate of the participants who succeeded in activating the app (359/481, 74.6% with the more seamless user journey vs 328/608, 53.9% with the original user journey).

#### Configurable Reminders and Push Notifications

**Figure 5:**
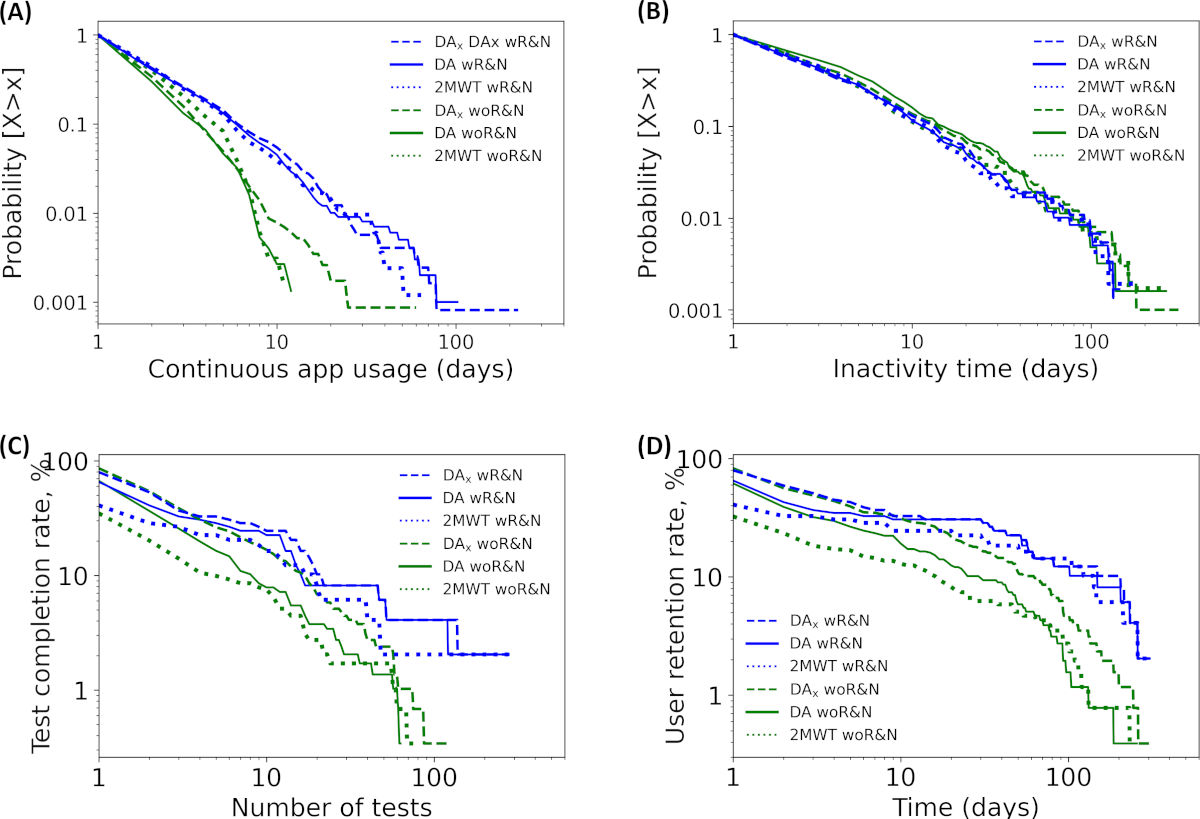
Complementary cumulative distributions of the continuous app use time (A), inactivity time (B), test completion rate (C), and user retention rate (D) of participants with multiple sclerosis (MS) using the Floodlight Open app with reminders and notifications (wR&N; blue) and without reminders and notifications (woR&N; green) for all daily activities performed in the fixed sequence (DA), all daily activities performed in the fixed sequence without Static Balance Test and U-Turn Test (DAx), and Two-Minute Walk Test (2MWT). Continuous app usage time, test completion rate, and user retention rate improved with reminders and notifications after an initial onboarding phase, whereas inactivity time was comparable between the 2 cohorts.

The analysis on configurable reminders and push notifications included 172 participants with MS to whom this UX design feature was available and 350 participants with MS to whom this feature was not available (Table 3). The cumulative distributions of the continuous usage times of participants with MS using the Floodlight Open app with reminders and notifications and those using the Floodlight Open app without reminders and notifications are plotted in a log-log scale for DA, DA_x_, and 2MWT in Figure 5A. The introduction of reminders and notifications increased the continuous app usage times. The longest continuous usage times with reminders and notifications versus those without reminders and notifications were 103 versus 13 days for DA, 224 versus 61 days for DA_x_, and 65 versus 12 days for 2MWT. Similarly, reminders and notifications increased the proportion of participants using the app for at least 3 consecutive days (DA and DA_x_: ~30% vs ~12%; 2MWT: ~30% vs ~20%).

Figure 5B presents the inactivity time distributions from participants using the Floodlight Open app with configurable reminders and push notifications and those using the Floodlight Open app without configurable reminders and push notifications on a log-log scale for DA, DA_x_, and 2MWT. These distributions were similar for participants with reminders and notifications and those without reminders and notifications for DA, DA_x_, and 2MWT, exhibiting an approximate power law with a coefficient of 0.96 in both cohorts. The longest inactivity times with reminders and notifications versus without reminders and notifications were 145 versus 263 days for DA, 138 versus 309 days for DA_x_, and 181 versus 263 days for 2MWT.

Figure 5C presents the test completion rate distributions for DA, DA_x_, and 2MWT. These distributions show that the introduction of reminders and notifications resulted in higher test completion rates, except during the initial onboarding phase when DA and DA_x_ completion rates were similar in both cohorts. During this onboarding phase, day-1 test completion rates with and without reminders and notifications were 66% (113/172) and 66.9% (234/350), respectively, for DA and 81.3% (140/172) and 86% (301/350), respectively, for DA_x_. These test completion rates are expected to be <100% because not all participants who activated the Floodlight Open app proceeded to perform at least 1 DA or DA_x_ series. However, the effectiveness of introducing reminders and notifications became evident after this initial onboarding phase (ie, after the first DA series and after the third DA_x_ series; Figure 5C). When considering the 2MWT instead, participants with reminders and notifications showed higher test completion rates than participants without reminders and notifications (41.5% [72/172] with reminders and notifications vs 34.8% [122/350] without reminders and notifications) on the first day itself. Across all 3 test series, the largest number of test series completed increased with the introduction of reminders and notifications (279 vs 64 for DA, 283 vs 126 for DA_x_, and 302 vs 76 for 2MWT).

Figure 5D presents the user retention rate distributions. During the initial onboarding phase, user retention rates were comparable between participants with reminders and notifications and those without reminders and notifications. On day 1, DA user retention rates were 65.7% (113/172) and 61.7% (216/350), respectively, and DA_x_ user retention rates were 79.6% (137/172) and 82.8% (290/350), respectively (Table 4). DA_x_ user retention rates continued to be comparable between both cohorts for approximately the first 10 days. After this onboarding phase, however, the effectiveness of reminders and notifications in improving user retention rate became evident. Interestingly, after the high drop-off observed during the first few days after the activation of the app (3 days for DA and DA_x_ and 2 days for 2MWT), all user retention rate distributions obtained with reminders and notifications plateaued up to approximately day 30. On day 30, user retention rates were considerably higher in participants with versus participants without reminders and notifications (53/172, 30.8% vs 34/350, 9.7% for DA; 53/172, 30.8% vs 60/350, 17.1% for DA_x_; and 39/172, 22.6% vs 22/350, 6.2% for 2MWT; Table 5).

**Table 5:** User retention rates of the Floodlight Open app with and without reminders and notifications from participants with MS^a^ and average user retention rates for medical apps and health and fitness apps reported by Rosenfelder [31] on days 1, 3, 7, 14, and 30.

Apps, configuration, and series			User retention rate				
			Day 1	Day 3	Day 7	Day 14	Day 30
**Floodlight Open app, % (n/N)**							
	**Without configurable reminders and push notifications**						
		DA^b^	61.7 (216/350)	32 (112/350)	23.4 (82/350)	16.3 (57/350)	9.7 (34/350)
		DA_x_^c^	82.8 (290/350)	48.8 (171/350)	34.5 (121/350)	28 (98/350)	17.1 (60/350)
		2MWT^d^	32.34 (113/350)	18.2 (64/350)	14.2 (50/350)	11.3 (39/350)	6.2 (22/350)
	**With configurable reminders and push notifications**						
		DA	65.7 (113/172)	36.6 (63/172)	32.6 (56/172)	30.8 (53/172)	30.8 (53/172)
		DA_x_	79.6 (137/172)	51.1 (88/172)	36.6 (63/172)	30.8 (53/172)	30.8 (53/172)
		2MWT	40.7 (70/172)	32.6 (56/172)	28.5 (49/172)	24.4 (42/172)	22.6 (39/172)
**Rosenfelder [31], 2020, %^e^**							
	**Medical apps**						
		Active	20	11.52	9.24	7.19	5.46
	**Health and fitness apps**						
		Active	18.37	10.56	7.77	5.56	3.6

^a^MS: multiple sclerosis.

^b^DA: all daily activities that were administered in a predefined, fixed sequence.

^c^DA_x_: all daily activities that were administered in a predefined, fixed sequence, except the Static Balance Test and U-Turn Test.

^d^2MWT: Two-Minute Walk Test.

^e^The absolute number of uses retained at each time point were not reported in Rosenfelder [31], 2020.

## Discussion

### Principal Findings

Smartphones enable out-of-clinic assessments of chronic neurological diseases. Despite the rapidly increasing number of mobile health care apps available for consumers’ self-care, there is a paucity of research into the UX of DHTTs for MS. In this paper, we present our qualitative and quantitative UX analyses of the Floodlight Open app and demonstrate that the adoption of key UX design features markedly improved user acquisition, engagement, and retention. Understanding how the presence or absence of specific UX design features affects participants’ experiences offers important guidance for the refinement of an existing or the design of a new DHTT. Therefore, UX design features are a significant consideration when designing and evaluating such tools.

Assessing MS symptoms over time with the Floodlight Open app requires specific user stimulation and guidance through seamless and flexible user journeys enhanced by engaging features to improve the overall UX. By considering participants’ feedback collected in the quantitative UX substudy, we enhanced the onboarding process by implementing a more seamless user registration and app activation journey and improved the UX through configurable reminders and push notifications. We derived several distributions from real-world data that describe key aspects of participants’ behavior in their environment. Such measurements and their statistical analysis might be used to help describe realistic user behavior and design health care apps similar to our Floodlight Open app. Our findings allow us to gain a better understanding of users’ experience in such settings and to make informed design decisions for self-monitoring mobile apps that support people with MS.

Users’ first experience with the registration to the Floodlight Open study and the activation of the study app is important to increase user acquisition. Furthermore, the first few days after activating the app are similarly critical for long-term engagement and retention. For instance, user acquisition, engagement, and retention can all be improved by guiding users throughout the Floodlight Open study registration, app activation process, and daily activities, reminding them about the daily activities to be performed and allowing them to plan when to perform the daily activities. Our results demonstrate that a seamless UX is essential to minimize the rate at which users drop out along the end-to-end journey. With a seamless user journey, users are led along the registration-activation journey and are not distracted by unrelated content or activities, resulting in a higher conversion rate from creating an account to activating the Floodlight Open app.

Configurable reminders and push notifications can help keep the participants engaged with mobile health care apps, such as the Floodlight Open app, and help retain them beyond the initial onboarding phase. Our findings show that continuous use times, test completion rates, and user retention rates are generally higher with configurable reminders and push notifications than without configurable reminders and push notifications, whereas inactivity time is comparable between the 2 cohorts. This improvement in user engagement is most evident for 2MWT from the first day and for DA and DA_x_ after the initial onboarding phase, during which the participants are still exploring the Floodlight Open app. Therefore, to fully optimize the UX, the app should ask the users to accept and receive reminders and notifications only after this initial onboarding phase [32]. Interrupting users with notifications too soon after the activation of the app may create frustration and may ultimately lead them to abandon the app. The app should, therefore, minimize the mental and physical interaction efforts during the first few days of use.

Comparing the different test series, we noted slightly worse user retention for 2MWT than for either DA or DA_x_. Taking the 2MWT requires the greatest effort, which is in line with some dissatisfaction reported in a previous proof-of-concept study [33]. It is possible that the study participants did want to take the 2MWT but did not find a suitable opportunity to take it each day. In fact, participants in Floodlight Open were found to persistently take the 2MWT at least once per week [15], which suggests that a more flexible or less frequent assessment schedule could be beneficial.

### Comparison With Prior Work

Although several other smartphone apps are available for people with MS (for a review on MS apps, refer to the study by Howard et al [34]), to our knowledge, this is the first analysis to assess the impact of UX design features on user engagement and retention. Nonetheless, previous analyses have shown that user engagement and retention remain two of the barriers to the long-term successful deployment of DHTTs (for reviews, refer to works of Pratap et al [6] and Amagai et al [35]). Several UX design features have been suggested to improve the overall UX, including a more seamless user journey, configurable reminders, and push notifications [23,35-38]. However, differences in the definitions used to assess user retention (eg, user retention definitions based on the user simply opening or interacting with the DHHT [31] vs completing a test or series of tests [6]), limited statistical power due to small sample sizes [37], and differences in the observed study period [37] may make direct comparisons of user retention across different DHHTs challenging.

To compare like with like, we computed the user retention with the Floodlight Open app for the same observation period as that reported in the study by Rosenfelder [31] for medical as well as health and lifestyle apps. Both data sets indicate that the largest reduction of users occurs from days 1 to 3, whereas the reduction in user retention is low between days 7 and 30. This suggests that a significant proportion of users who will not use the Floodlight Open app (or other health care apps) in the long term will stop using the app within the first 3 days and that this dropout is likely to be observed within the first 7 days. These results also suggest that participants who use the app for >3 days are likely to use it for at least 30 days. A similar dropout during the first 7 days has also been previously reported for the whole study cohort of Floodlight Open [15].

Of note, our user retention rates are approximately 2- to 6-fold higher than those reported by Rosenfelder [31] (Table 5) despite using a more conservative definition of user retention (completing a test series vs simply interacting with or opening the app). It is conceivable that not all apps included in the report by Rosenfelder [31] feature the same UX design features as the Floodlight Open app does. However, favorable user retention rates were observed with the Floodlight Open app even when the app did not have configurable reminders or push notifications. This suggests that differences in the perceived benefit of the app might have also contributed to the favorable retention profile, although other reasons cannot be excluded [39]. Despite our favorable user retention and user engagement findings, a previous analysis showed that improvement in smartphone sensor data collection could be achieved through passive data collection methods [15].

### Limitations

A few limitations are noted. First, while participants could turn off the configurable reminders and push notifications, this action was not logged. Instead, we compared participants who enrolled before with those who enrolled after these features were implemented. For the former cohort, we included only the data collected up to the implementation of reminders and notifications in our analyses to be able to assess the impact of reminders and notifications. Second, our results were influenced by the duration of the study and by incoming participants who activated the Floodlight Open app on different dates over the duration of the study and might not be part of the study for the same number of days. Besides, for events approaching the duration of the study, there is an artificially lower likelihood of observation, and events lasting longer than the study cannot be observed. Finally, the use of the Floodlight Open app in a clinical study other than the Floodlight Open observational study may boost user engagement and retention due to additional motivational incentives. Such clinical referrals have been shown to improve user retention [6]. However, information on which participants participated in such studies is not available, and without it, accounting for it is not possible.

### Conclusions

We presented the qualitative and quantitative UX analyses of the Floodlight Open app, a DHTT for MS. Learnings from the qualitative UX analysis led to the implementation of a more seamless user journey and the introduction of configurable reminders and push notifications. These US design features improved user acquisition, user engagement, and user retention. The continued assessment of UX and improvement of UX design features are critical steps in optimizing the long-term adoption of the Floodlight Open technology and similar DHTTs.

## Abbreviations

2MWT: Two-Minute Walk Test
DA: all daily activities that were administered in a predefined, fixed sequence
DaS: Draw a Shape Test
DAx: all daily activities that were administered in a predefined, fixed sequence excluding the Static Balance Test and U-Turn Test
DHTT: digital health technology tool
DMQ: Daily Mood Questionnaire
FLO ID: personal Floodlight Open unique identifier
IPS: Information Processing Speed
MS: multiple sclerosis
PT: Pinching Test
SBT: Static Balance Test
UTT: U-Turn Test
UX: user experience

## Data Availability

Request for the data underlying this publication requires a detailed, hypothesis-driven statistical analysis plan that is collaboratively developed by the requester and company subject matter experts. Such requests should be directed to dbm.datarequest@roche.com for consideration. Anonymized records for individual patients across >1 data source external to Roche cannot, and should not, be linked due to a potential increase in the risk of patient reidentiﬁcation.
